# Anti-Angiogenic Alternative and Complementary Medicines for the Treatment of Endometriosis: A Review of Potential Molecular Mechanisms

**DOI:** 10.1155/2018/4128984

**Published:** 2018-10-01

**Authors:** Weilin Zheng, Lixing Cao, Zheng Xu, Yuanyuan Ma, Xuefang Liang

**Affiliations:** ^1^Guangzhou University of Chinese Medicine, China; ^2^Team of Application of Chinese Medicine in Perioperative Period, Guangdong Provincial Hospital of Chinese Medicine, China; ^3^Department of Gynecology, Anyang Hospital of Traditional Chinese Medicine, China; ^4^Department of Gynecology, Guangdong Provincial Hospital of Chinese Medicine, China

## Abstract

Endometriosis is caused by the growth or infiltration of endometrial tissues outside of the endometrium and myometrium. Symptoms include pain and infertility. Surgery and hormonal therapy are widely used in Western medicine for the treatment of endometriosis; however, the side effects associated with this practice include disease recurrence and menopause, which can severely influence quality of life. Angiogenesis is the main biological mechanism underlying the development of endometriosis. Numerous natural products and Chinese medicines with potent anti-angiogenic effects have been investigated, and the molecular basis underlying their therapeutic effects in endometriosis has been explored. This review aims to describe natural products and compounds that suppress angiogenesis associated with endometriosis and to assess their diverse molecular mechanisms of action. Furthermore, this review provides a source of information relating to alternative and complementary therapeutic products that mediate anti-angiogenesis. An extensive review of the literature and electronic databases, such as the China National Knowledge Infrastructure, PubMed, and Embase, was conducted using the keywords ‘endometriosis,' ‘traditional Chinese medicine,' ‘Chinese herbal medicine,' ‘natural compounds,' and ‘anti-angiogenic' therapy. Anti-angiogenic therapy is an emerging strategy for the treatment of endometriosis. Natural anti-angiogenic products and Chinese medicines provide several beneficial clinical effects, including pain relief. In this review, we summarize clinical trials and experimental studies of endometriosis using natural products and Chinese medicines. In particular, we focus on anti-angiogenic products and alternative and complementary medicines for the treatment of endometriosis and additionally examine their therapeutic efficacy and mechanisms of action. Anti-angiogenic natural products and/or compounds provide a new approach for the treatment of endometriosis. Future work will require randomized trials with larger numbers of subjects, as well as long-term follow-up to confirm the findings described here.

## 1. Introduction 

Endometriosis is a gynecological disease in which functional endometrial glands and stroma are implanted outside of the uterine cavity. This is a common benign disease in women of reproductive age [1, 2]. The main symptoms of endometriosis are dysmenorrhea, endometriosis-associated pain, abdominal pain, and infertility [3, 4]. High levels of anxiety and depression are known to amplify the severity of pain [5]. Various hypotheses have been proposed to explain the pathology of endometriosis and associated pain; however, the biological basis for these processes remains unclear. Endometriosis is increasingly regarded as a complex multi-factorial condition of uncertain etiology, in which the immunological, hormonal, and genetic environment contribute to the disease. The adhesion and proliferation of endometrial tissue, cellular invasion, and neoangiogenesis are essential elements in the pathogenesis of endometriosis.* In vitro *and* in vivo* evidence suggests that pathogenesis may be related to abnormalities in nerve fibers, abnormal expression of nerve growth factors, and/or abnormal expression of inflammatory factors. Recent studies also suggest a link between angiogenesis and the growth of new nerve fibers, which together affect endometriosis associated pain [6]. Neurovascular formation may be an important component and/or cause of endometriosis.

At present, endometriosis is difficult to cure, with only symptomatic forms of treatment available. These main treatments include surgery, drug treatment, and long-term comprehensive individual treatment [7]. Traditional medical therapies are nonsteroidal anti-inflammatory drugs (NSAIDs), as well as the suppression of ovarian function by hormonal drugs such as androgens, aromatase inhibitors, selective progesterone receptor modulators, oral contraceptives, danazol, gestrinone, gonadotropin-releasing hormone (GnRH) agonists, and dienogest [8]. GnRH antagonists, aromatase inhibitors, selective progesterone receptor modulators, anti-tumor necrosis factor-*ɑ* (TNF-*ɑ*), and anti-angiogenic factors are new treatments for endometriosis [9, 10]. The medical treatment of endometriosis also results in several side effects [7]. It is important to note that although surgical resection (non-radical surgeries) completely removes all visible ectopic lesions and improves fertility, recurrence may occur and pain may not be completely relieved [11]. Consequently, the exploration of new complementary and alternative medicines is important to potentially improve treatment outcomes for patients with endometriosis.

Complementary and alternative medicines may provide new strategies for the management of chronic pelvic pain associated with endometriosis. Several anti-angiogenesis treatments have been shown to shrink lesions, loosen pelvic tissue adhesions, reduce pelvic congestion, relieve pelvic pain, and improve ovarian function [12]. In endometriosis, anti-angiogenesis is associated with multiple genes, factors, and signaling pathways. Herein, we will focus on the major molecular targets of Chinese herbal medicines and the bioactive molecules present in these medicines that contribute to the anti-angiogenic therapy; in doing this, we hope to identify new forms of treatment for endometriosis.

## 2. Methods

A literature search was performed in China National Knowledge Infrastructure, PubMed, and Embase for original articles written in English or Chinese and electronically listed before May 2018. The search included the following key words: ‘endometriosis,' ‘angiogenesis,' ‘anti-angiogenic,' ‘angiogenesis inhibitor,' ‘ectopic endometrium,' ‘natural product,' ‘natural compound,' ‘Chinese medicine,' ‘alternative and complementary,' and ‘Chinese medical practitioners.' Basic and clinical research, and* in vivo* and* in vitro* studies were included. However, it is important to note that this study incorporates research based on basic research and is not a systematic review.

## 3. Results

### 3.1. Angiogenic Mechanisms in Endometriosis

The mechanistic basis for endometriosis is most likely to represent a complex network process that includes immune responses [13], inflammatory responses, hormones, pelvic adhesions, angiogenesis, and neural mechanisms. Angiogenesis is a multistep and intricate process of new blood vessel formation that involves the extravasation of growth factors and degradation of the extracellular matrix, as well as migration, proliferation, and tube formation by endothelial cells [14, 15]. Endometriosis is a complex disease that is absolutely dependent on the development of new blood vessels and the association of numerous angiogenesis-related factors [16]. These include vascular endothelial growth factor (VEGF), vascular endothelial growth factor receptor (VEGFR), angiopoietin (Ang)-Tie2 axis, and delta-like 4 (Dll4)-Notch signal pathways [17].

VEGF and its receptor, VEGFR, are powerful angiogenic factors that play important roles in tumor formation and other angiogenesis-associated diseases. VEGF affects endothelial cell proliferation, migration, and permeability [18, 19]. The peritoneal fluid levels of VEGF in patients with type IV endometriosis are known to be higher than those with type I and type II endometriosis, as well as in patients without endometriosis [20]. Therefore, VEGF is an important biomarker for endometriosis [21, 22]. VEGFC acts on endothelial cells and promotes early angiogenic responses through the VEGFR2-mediated pathway, which promotes endothelial function and endometriosis vascular permeability [23, 24].

Furthermore, immune and inflammatory proteins are closely linked to angiogenesis [25]. Numerous factors are involved in endometriosis-associated angiogenesis including matrix metalloproteinases (MMPs), TNF-*ɑ*, cyclo-oxygenase (COX), and hypoxia-inducible factor 1*α* (HIF-1). MMPs degrade the extracellular matrix and are key components of endometrial adhesion and angiogenesis [26, 27]. TNF-*ɑ* promotes angiogenesis during the progression of endometriosis. There is a strong relationship between the TNF-*α* gene promoter region -1031T/C polymorphism and endometriosis [28]. COX inhibitors prevent endometrium implantation to ectopic sites and induce regression of established endometriotic lesions [29]. Prostaglandin E2 (PGE2) significantly increases MMP-2 activity, while the inhibition of COX-2 and/or phosphorylated protein kinase B(AKT) suppresses MMP-2 activity in human endothelial cells [30]. Toll like receptor-4 (TLR4) is distributed on vascular endothelial cells and endometrial epithelial cells, where it regulates angiogenesis and activates inflammatory responses [31]. Nuclear factor-kappa B (NF-*κ*B) may also be involved in the endometrial biological alterations associated with endometriosis [32]. These factors either directly or indirectly affect endometriosis-associated angiogenesis.

### 3.2. Anti-Angiogenic Treatment for Endometriosis

Neuro-angiogenesis and angiogenesis are essential for the development of endometriosis. Anti-angiogenic compounds may inhibit early-stage or post-surgical endometriotic lesions [33]. Numerous drugs have been shown to exert anti-angiogenic effects. Anti-angiogenic substances include growth factor inhibitors, endogenous angiogenesis inhibitors, COX-2 inhibitors, phytochemical compounds, immunomodulators, dopamine agonists, peroxisome proliferator-activated receptor agonists, progestins, GnRH agonists, and angiogenesis inhibitors [34].

The role of*** GnRH analogues***  has been shown to induce apoptosis and reduce angiogenesis by increasing the expression of Bax and Fas-ligand (FasL) and decreasing Bcl-2, VEGF-A and interleukin-1 beta (IL-1*β*) in eutopic endometrial cell cultures [35].*** Dienogest***  is a novel progestin that is highly selective for progesterone receptors and inhibits endometriosis. Dienogest has been shown to reduce proliferation, aromatase expression and angiogenesis, and increase apoptosis in human endometriosis cells [36].

***Angiogenesis inhibitors***  are substances that antagonize or inhibit the development of new blood vessels, and target angiogenesis-related targets. These inhibitors specifically target either angiogenic factors or tyrosine kinases involved in the regulation of angiogenic pathways. Bevacizumab is a humanized anti-VEGF monoclonal antibody used in the treatment of cancer. There is some evidence to show that VEGFR-1 may cross-talk with VEGFR-2 and initiate the signaling cascades described above. The anti-VEGF antibody, bevacizumab, was as effective as GnRH agonists in the regression of endometriotic lesions in a rat endometriosis model, and significantly inhibited cell proliferation in lesions, reduced vascular density, increased the proportion of apoptotic cells, and reduced VEGF levels in the peritoneal fluid of female BALB/c mice [37–39]. Ranibizumab has been shown to significantly reduce the size of endometriotic implants and caused atrophy of such lesions in rats model by reducing explant levels of VEGF [40]. Thalidomide treatment was also shown to significantly reduce the levels of VEGF-A and myeloperoxidase (MPO) in rats model [41, 42]. Sunitinib, an anti-endometriotic agent, was also shown to reduce the cross-sectional area of endometriotic cysts by 78.8% and caused complete cyst disappearance in 50% of rats, although the TUNEL assay showed evidence of increased levels of apoptosis [43–45].

***Statins,*** known as 3-hydroxy-3-methyl-glutaryl-coenzyme A reductase (HMG-CoA reductase) inhibitors, are a class of lipid-lowering medications and represent a promising drug for the treatment of endometriosis because they have anti-proliferative, anti-angiogenic, antioxidant, and anti-inflammatory properties and can inhibit matrix metalloproteinase activity [46–48]. Simvastatin has been shown to markedly inhibit tumor angiogenesis in human colorectal cancer and breast cancer by reducing the levels of VEGF and hypoxia-inducible factor-1*α*(HIF-1*α*) [49, 50]. Simvastatin is used for the clinical treatment of endometriosis-related pain and relapse pain; a previous study showed that there was no significant difference in the efficacy of simvastatin and GnRHa when compared between these two patient groups [51].

***Dopamine and its agonists,***  such as cabergoline (Cb2), decreased in the percentage of active endometriotic lesions and cellular proliferation index in female nude mice implanted human endometrium fragments. Activation of the dopamine (Dp)/dopamine receptor 2 (Dp-r2) pathway was involved in the VEGF/VEGFR-2 signaling process and can reduce neoangiogenesis [52]. Cabergoline also inhibited the growth of established endometriotic lesions in a rat endometriosis model. Its anti-angiogenic action is mediated by inhibition of VEGFR-2 phosphorylation [53]. Compared to luteinizing hormone releasing hormone (LHRH) agonist, cabergoline (dostinex) yields better resulted in decreasing the size of endometrioma by vaginal ultrasound in endometriosis patients in a prospective randomized study [54]. Quinagolide, another non-ergot-derived dopamine agonist, reduced the levels of both IL-6 and VEGF in peritoneal fluid and was shown to be of potential use for the treatment of endometriosis-induced endometriosis in a rat model [55]. These drugs may regulate the angiogenesis process by regulating the regulation of related immune inflammatory factors and angiogenic factors.

A range of* in vitro* and* in vivo* studies of several potential drugs that reduce angiogenesis and endometriotic implant size have been reported in mice ([29, 56–58]), rats ([59–61]), primates ([62]), and human cell culture ([63]).** See **Table 1** for a summary.**

Endometriosis research focuses upon reducing the side effects of treatment as well as understanding the mechanistic basis for the therapeutic effects of these treatments. Anti-angiogenic drugs represent a new direction for the treatment of endometriosis. However, clinical evidence for their efficacy and safety is not yet available. Most anti-angiogenic drugs show efficacy in preclinical models (as well as for the treatment of cancer), but are generally not useful in the clinic. Angiogenesis inhibitors show promise in the treatment of endometriosis, but these anti-angiogenic agents are effective in inhibiting pathological angiogenesis; they also have serious side effects that preclude their use in many patients. For example, bevacizumab may have potential adverse effects such as proteinuria, hypertension, thromboembolism, and hemorrhage [64]. Angiogenesis inhibitors have also been associated with cardiovascular toxicities, such as hypertension, left ventricular systolic dysfunction, heart failure, and conduction abnormalities [65, 66]. Anti-angiogenic and potentially damaging effects on fetal development and ovarian dysfunction appears to be a plausible side effect of the angiogenic treatment in cancer therapy [67]. The effects of long-term use upon side effects, ovarian reserve function, and pregnancy safety for women's issues and other issues require further study. Therefore, there is a need to identify more effective and safer anti-angiogenesis drugs.

### 3.3. Natural-Product Angiogenesis Inhibitors for Endometriosis

Some plants and their active compounds/natural products, in combination with standard therapies, may improve medical treatment for endometriosis. These may include Ayurvedic products, homeopathic products, dietary modifications, and herbal therapies. Many of these are phytochemicals that have been shown to have anti-cancer and anti-angiogenic effects. These include polyphenols, flavonoids, alkaloids, terpenoids, and tannins as antioxidants. Melatonin, resveratrol, xanthohumol, and epigallocatechin-3-gallate have been reported as new and effective compounds for the long-term treatment of endometriosis [68–70].** Please refer to **Table 2** for a list of natural-product angiogenesis-inhibitors for endometriosis.**

***Epigallocatechin Gallate (EGCG)*,**  which is derived from green tea, has powerful anti-angiogenic properties. It may be effective against endometriosis through inhibition of angiogenesis, adhesions, and invasion by endometriotic lesions on an endometriosis mouse model transplanting human eutopic endometrium [71]. EGCG selectively inhibits the expression of vascular endothelial growth factor VEGF-C (VEGFC) and the tyrosine kinase receptor, VEGFR2, by inhibition of the VEGFC/VEGFR2 signaling pathways, which inhibits endometriosis-associated angiogenesis of endothelial cells [72]. EGCG also could inhibit transforming growth factor (TGF)-*β*1-stimulated activation of mitogen-activated protein kinase (MAPK) and Smad signaling pathways in endometrial and endometriotic stromal cells [73]. EGCG also significantly inhibited the activation of the MAPK/Smad signaling pathways in TGF-*β*1-stimulated endometrial and endometriotic stromal cells. EGCG and prodrug of green tea epigallocatechin-3-gallate (pro-EGCG) significantly reduced the size and weight of endometriotic lesions via the induction of apoptosis [74]. Pro-EGCG (EGCG octaacetate), a prodrug of EGCG, utilized to enhance the stability and bioavailability of EGCG in vivo. The inhibitory effect of pro-EGCG was greater than that of EGCG in mice model [75]. In vitro and in vivo studies have shown that resveratrol and EGCG inhibit the growth and survival of ectopic endometriosis tissue [76]. EGCG and pro-EGCG could represent a potent anti-angiogenesis agent for endometriosis.

***Resveratrol***,  3,4,5-trihydroxy-trans-stilbene, a phytoalexin derived from grapes and other food products, is a plant-derived polyphenolic phytoalexin that inhibits angiogenesis in peritoneal and mesenteric endometriotic lesions, significantly reduces micro-vessel density, inhibits proliferation, and increases apoptosis [77, 78]. Furthermore, resveratrol has been shown to reduce* in vitro* invasiveness of endometriotic stromal cells (ESCs) and suppress the inflammatory response [79]. Resveratrol has also been shown to significantly reduce histopathological grade, as well as the expression of matrix metalloproteinases-2 (MMP-2), matrix metalloproteinases-9 (MMP-9), and VEGF in a rat model of endometriosis; this drug also reduced levels of interleukin-6 (IL-6), IL-8, and TNF-*ɑ* levels in plasma and peritoneal fluid [80]. Resveratrol was also shown to significantly reduce the peritoneal fluid levels of VEGF and monocyte chemoattractant protein-1 (MCP-1) in an experimental rat model [81]. Resveratrol could act as both an agonist and antagonist of estrogen and reduce human endometrial proliferation through estrogen receptor-*ɑ* (ESR*ɑ*) [82]. Resveratrol was also shown to potentiate the inhibitory effects of simvastatin on cholesterol biosynthesis and 3-hydroxy-3-methylglutaryl-CoA reductase (HMGCR) enzyme activity while also abrogating the stimulatory effect of simvastatin on HMGCR mRNA transcripts and protein expression in human endometrial stromal (HES) cells [83].

Resveratrol has potent antioxidant properties [78], anti-inflammatory effects [77], an enhanced capacity to induce apoptosis in endometriotic stromal cells [84], as well as anti-angiogenic effects through the inhibition of vascularization and cell proliferation [81]. Resveratrol could reduce the pain scores and the level of carcinoembryonic antigen (CA125) in the treatment of endometriosis-associated pain patients in randomized clinical trials. However, the effects of resveratrol combined with the combined oral contraceptive (COC) were not superior to those of COC alone after 42 days of treatment [85].

***Palmitoylethanolamide *(PEA)** is an endogenous fatty acid amide that has anti-inflammatory and neuro-protective effects. Co-micronized palmitoylethanolamide/polydatin (PEA/PLD) inhibited the development of endometriotic lesions in a rat model of surgically induced endometriosis. PEA/PLD could also decrease angiogenesis (VEGF), nerve growth factor (NGF), intercellular adhesion molecule (VCAM-1), MMP-9, and lymphocyte accumulation, and reduce peroxynitrite formation (poly-ADP) ribose polymerase activation, Ik-B*ɑ* phosphorylation, and NF-*κ*B translocation [86].

***Achillea biebersteinii Afan.***:  the aerial components of* A. biebersteinii* were collected from the Beynam Forest. The therapeutic effect of an ethyl acetate (EtOAc) extract of* A. biebersteinii* was attributed to the flavonoid aglycones found in the extract. This EtOAc extract reduced the post-treatment volumes of endometrial foci in non-pregnant Sprague Dawley rats and reduced levels of TNF-*α*, VEGF, and IL-6 in the peritoneal fluid [87].

***Viburnum opulus L***.:  the fruits of* V. opulus* L. (Wu Beizi) have been used to treat endometriosis, primary and secondary dysmenorrhea, and ovarian endometriosis. Active extracts were analyzed by high-performance liquid chromatography (HPLC), and chlorogenic acid was found to be the main active compound. Both EtOAc and methanol(MeOH)extracts of* V. opulus *L. showed significant inhibition of TNF-*α*, VEGF, and IL-6 levels in a rat model of surgically induced endometriosis [88].

***Flavonoids***  are a class of secondary plant and fungus metabolites that includes flavones, flavonols, flavanones, anthocyanins, and isoc acid was found to be the main active compound. Over 5000 naturally occurring flavonoids have been characterized from various plants. Flavonoids have anti-angiogenic properties in several diseases and may have potential for the treatment of endometriosis [89, 90].

***Genistein***  is a type of isoflavone. Isoflavones have been shown to exhibit anti-microbial, anti-mutagenic, anti-oxidant, anti-inflammatory, and anti-angiogenic activities [91]. Doses of genistein were shown to significantly reduce the expression of estrogen receptor *α* in mice, increase the expression of estrogen receptor *β*, and decrease the expression of VEGF and HIF-1*α* in peritoneal tissues using immunohistochemical techniques, reaching levels comparable to that of a group of mice treated with leuprolide acetate; in this study, genistein also regulated inflammation and angiogenesis by modulating inhibition upon the estrogen receptor in a murine model of peritoneal endometriosis [92].

***Xanthohumol***  is a natural product found in the female inflorescences of humulus lupulus. Xanthohumol could act as a pleiotropic chemopreventive agent for cancer due to its anti-proliferative, anti-inflammatory, and anti-angiogenic properties. For example, xanthohumol was shown to effectively reduce the size of cancerous lesions and reduce the level of phosphoinositide 3-kinase protein (PI 3-kinases) in a BALB/c mouse model of endometriosis; additional analysis showed that treatment with xanthohumol could significantly decrease the microvessel density (MVD) and did not affect the histomorphology, proliferation, or vascularization of the uterine horn and ovary during such treatment [93].

***Sea buckthorn and St. John's wort***  Sea buckthorn (*Hippophae rhamnoides L.*) and St. John's wort (*Hypericum perforatum L.*) are used for the treatment of uterus inflammation and endometriosis. A mixture of sea buckthorn and St. John's wort oils (HrHp oil) significantly decreased the volumes of endometriotic implants and reduced the levels of TNF-*α*, vascular VEGF and IL-6 in peritoneal fluids in a surgically induced endometriosis rat model [94].

#### 3.3.1. Other Potential Anti-Angiogenic Natural Compounds

Numerous plant-based products are known to have anti-inflammatory, anti-proliferative, analgesic, antispasmodic, and antioxidant properties. As such, these products represent potential treatments for endometriosis and provide a source of drugs for future anti-angiogenesis studies of endometriosis.

Flavonoid natural compounds may represent a particularly good potential source of drugs for endometriosis. Naringenin belongs to the family of flavanones that includes six categories of flavonoids. Naringenin is a phytoestrogen that exhibits estrogen and antiestrogenic activity and is mainly extracted from citrus fruits. This phytoestrogen could suppress proliferation and increase apoptosis via depolarization of mitochondrial membrane potential and the generation of reactive oxygen species (ROS) in human endometriosis cell lines (VK2/E6E7 and End1/E6E7) [95]. Naringenin also could inhibit of angiogenesis by regulating VEGF/Kinase insert domain receptor (vascular endothelial growth factor receptor 2, KDR, VEGFR2) signaling pathway in human endothelial cells [96]. Mitogen-activated protein kinases (MAPKs) are a widely distributed form of protein kinase containing serine and threonine residues in the cytoplasm. Nobiletin has also been shown to exhibit anti-angiogenic activity* in vivo* in a zebrafish model and* in vitro* in a human umbilical vein endothelial cell (HUVEC) model by blocking the VEGFA/VEGFR2-MAPKs signaling pathway [97]. However, whether naringenin can affect the development of endometriosis by regulating angiogenesis requires further study.

In addition,***Kushecarpin D* (KD)**,  a novel flavonoid isolated from the traditional Chinese herbal medicine Kushen (the dried root of* Sophora flavescens* Ait) exerted anti-angiogenic effects by inhibition of cell proliferation, cell migration, cell adhesion, and tube formation [98].* Marsdenia tenacissima *could inhibit the proliferation of human umbilical vein endothelial cells (HUVECs) by blocking cell cycle progression from G1 to S phases and by blocking migration and tube formation in HUVECs [99].* Wild Chrysanthemum and Uniflower Swiss Centaury *Root have also been shown to have anti-angiogenic effects in zebrafish by influencing both pro-angiogenic mechanisms and negative regulation of angiogenesis [100]. Saikosaponin C is one of the saikosaponins found in* Radix bupleuri* and may also have potential therapeutic effects against angiogenesis [101]. Sulfated polysaccharides/glycopeptides and flavonoids may synergize with anti-angiogenic drugs by inhibiting basic fibroblast growth factor** (**bFGF), HIF-1*α*, and the VEGF signal pathway [102].

Recently, a growing number of natural products have been shown to possess anti-angiogenic activities with multiple molecular mechanisms of action. Anti-angiogenic therapy using natural compounds has been extensively studied in tumors and angiogenic diseases. Endometriosis features tumor-like angiogenic behavior and the related targets and signaling pathways are therefore similar. Anti-angiogenic natural compounds could also represent a potential treatment for endometriosis. However, for many of these compounds, the mechanism of action is unclear and clinical trials have not yet been completed. Furthermore, the safety and pharmacotoxicity of these compounds also need further evaluation. Natural compounds therefore require significant development for medical application.

### 3.4. Anti-Angiogenic Agents Isolated from Chinese Herbal Medicines

Chinese herbal medicine (CHM), which comprises a variety of compounds and has a long history of use in China, is emerging as a treatment choice for numerous therapeutic applications, including anti-angiogenesis. Many Chinese herbs and formulas promote blood circulation, reduce blood stasis, and have anti-platelet effects, thus eliciting anti-angiogenic effects. Blood-activating and stasis-resolving herbals, tonifying qi herbals, as well as heat-clearing and detoxifying herbals, have anti-angiogenic effects, and their mechanism of action is related to the regulation of angiogenesis-related factors and signaling pathways. Due to the complexity of Chinese herbs, the active ingredients of such herbs are of increasing interest for medical application. The core compounds of traditional Chinese medicine may represent the key to pharmacological action. Due to the large number of compounds in traditional Chinese medicine, we only focus here on the main pharmacodynamic compounds identified to date.** See **Table 3** for a list of Chinese herbs commonly used to treat blood circulation problems associated with endometriosis.**

***Curcumin and Curcuma longa *(Jianghuang)**:  a rhizomatous plant of the ginger family has been associated with profound anti-inflammatory and antioxidative functions for centuries. Curcumin is also the active ingredient in the turmeric plants* Rhizoma curcumae* (E'zhu),* Rhizoma sparganii* (Sanleng), and* Curcuma rcenyujin* (Yujing) and a variety of traditional Chinese herbs. Several studies have demonstrated the anti-inflammatory, anti-oxidant, anti-tumor, anti-angiogenesis, and anti-metastatic activities of curcumin. The anti-angiogenic activity of curcumin has been assessed* in vivo and in vitro. *Curcumin has been found to decrease the MVD and VEGF protein expression in the heterotopic endometrium in a rat model of endometriosis [103]. Following treatment with curcumin, an increase the percentage of cells in G1 and decrease in the percentage of S phase cells were observed among human endometriotic stromal cells, along with the reduced expression of VEGF. Curcumin has been shown to reduce cell proliferation, human ectopic and eutopic stromal cell growth, and inhibit VEGF secretion in humans [104]. Furthermore, curcumin inhibited cell proliferation and caused cell apoptosis, and reduced inflammation through suppression of inflammatory cytokines expression. Treatment with curcumin alone and/or in combination with deferoxamine contributed to a reduction in implant size and cell proliferation in a rat endometriosis model [105]. Curcumin also induced apoptosis through a cytochrome-C-mediated mitochondrial pathway and in a p53 (a tumor suppressor gene)-dependent and -independent manner in a mouse model of endometriosis [106]. Curcumin could act as an inhibitor of NF-*κ*B and MMP-3 by influencing signaling pathways and increasing the secretion of associated proteins in human endometriotic stromal cells, thus mediating the regression of endometriosis [107]. Curcumin also has been shown to reduce estradiol (E2) production to suppress endometriosis endometrial cells [108]. Therefore, curcumin may influence inflammation, attachment, and angiogenesis in endometrial lesions, with potential benefit as a pharmacological agent and Chinese medicinal product for the prevention and treatment of endometriosis [109].

***Radix ginseng* (Renshen)**:  the dry root of* Panax ginseng C.A. Mey.* has been traditionally used as herbal medicine. The chemical components of ginseng include polysaccharides, ginsenosides, peptides, polyacetylene alcohols, and fatty acids. Ginsenoside Rg3 has various biological activities including immune enhancement, anti-oxidant, anti-inflammatory, neuro-protective, and anti-metabolic syndrome [110]. The anti-angiogenic effect of Rg3 is mainly due to the regulation of VEGF and suppression of the interaction between endothelial cells and the extracellular matrix by reducing the microvessel density in tumor tissues [111, 112]. Ginsenoside Rg3 has been shown to inhibit angiogenesis in endometrial lesions in a rat model by blocking VEGFR-2 and inhibiting the PI3K/Akt/mTOR signaling pathway [113]. Rg3 has been shown to effectively alter the fibrotic properties of human embryonic stem cells (HESCs) in patients with endometriosis, likely via miR-27b-3p modulation [114].

***Pueraria lobata* (Gegen) **  is a frequently used Chinese medicine. Puerarin, one of several known isoflavones, is derived from the root of* Pueraria lobata* (Gegen). It has been widely used for the treatment of cardiovascular and cerebrovascular diseases, diabetes, diabetic complications, Alzheimer's disease, and endometriosis [115]. Puerarin accelerated cardiac angiogenesis, improves cardiac function, and reduced myocardial infarction in a rat model by upregulating VEGFA, Angiopoietin-1(Ang-1), and Angiopoietin-2(Ang-2) [116].

*Puerarin*, a phytoestrogen with a weak estrogenic effect, binds to estrogen receptors and competes with 17-estradiol to produce an anti-estrogenic effect [117]. Puerarin inhibited the expression of aromatase cytochrome P450 (p450arom) and COX-2, thereby reducing the levels of E2 and PGE2 with concomitant inhibition of growth in the ectopic endometrium [118]. High levels of estrogen in the microenvironment of endometriotic lesions stimulated specific angiogenic growth factors such as VEGF, as well as other angiogenic factors. Puerarin suppressed estrogen-stimulated proliferation partly by downregulating the transcription of cyclin D1 and cdc25A by promoting the recruitment of corepressors to estrogen receptor-*α* as well as limiting that of co-activators in endometriotic stromal cells (ESCs) [119]. Another study showed that puerarin suppressed tissue invasion by ESCs and the vascularization of ectopic endometrial tissues stimulated by E2 [120].

***Salvia miltiorrhiza* (Danshen)**, a traditional Chinese herb, contains a number of compounds, including salvianolic acid (or salvianolic acid B), dihydrotanshinone, tanshinone I, and tanshinone II A, widely used as blood-activating herbals for a variety of diseases including cardiovascular disease. Tanshinone II A, which is a major diterpene quinone compound present in* S. miltiorrhiza*, induces angiogenesis by inducing the VEGF/VEGFR2 pathway and CD146 (melanoma adhesion molecule) both* in vitro* and* in vivo*. Tanshinone II A modulated angiogenic functions in HUVECs and dramatically suppressed VEGF-promoted cell migration and tube formation in human bone marrow-derived endothelial progenitor cells (EPCs) through the phospholipase C(PLC), protein kinase B(Akt), and c-Jun N-terminal kinase (JNK) signaling pathways. This is a promising natural product with potential for the treatment of cancer and other angiogenesis-related pathologies [121, 122].

Tanshinone II A, combined with the method of warming yang, removes blood stasis, effectively increasing serum p53 and p21 protein levels. Furthermore, this compound reduces the levels of murine double minute2 (MDM2), soluble intercellular adhesion molecule-1 (sICAM-1), soluble vascular cell adhesion molecule-1(sVCAM-1), and CA125 in patients with uterine endometriosis within three menstrual cycles [123]. Tanshinone II A could reduce cell viability, induce apoptosis, and inhibit cell migration and the invasion of ectopic endometrial stromal cells (EESCs). From a mechanistic standpoint, Tanshinone II A significantly reduced the expression of 14-3-3*ζ* in EESCs. The overexpression of 14-3-3*ζ* restores cell viability, migration, and invasion, but has no effect on apoptosis. The effects upon cell viability, migration, and invasion were shown to be mediated in a 14-3-3*ζ*-dependent manner, whereas the effects of apoptosis were mediated in a 14-3-3*ζ* independent manner [124].

Extracts of* S. miltiorrhiza* have also shown promise in treating endometriosis by markedly reducing the serum levels of CA125 and the levels of IL-18 and TNF-*α*, by significantly increasing the levels of IL-13 in the peritoneal fluid in a rat model [125]. One study showed that* S. miltiorrhiza* could inhibit ectopic stromal cell proliferation by inhibiting MMP-9 mRNA and protein expression [126]. Other active ingredients of* S. miltiorrhiza* may be involved in the anti-angiogenic process of endometriosis, but this requires further investigation.

***Paeonia lactiflora *Pall (Chishao)**  is a species of herbaceous. Paeoniflorin, the core component of* P. lactiflora.,* is commonly used in Chinese medicine prescriptions for gynecological diseases such as dysmenorrhea and irregular menstruation endometriosis. Common prescriptions such as Danggui Shaoyao and Danggui Sini decoctions are used for endometriosis-associated pain. Paeoniflorin has anti-inflammatory, analgesic, antipyretic, and platelet-inhibitory effects, and could influence the function of estrogen receptor-*α* (ESR*α*) and HIF-1*α* at network pharmacological and pharmacodynamic levels [127].

***Andrographis paniculata *(Chuanxinlian)**  is an annual herbaceous plant in the family Acanthaceae and has been traditionally used to treat infections and some diseases. Andrographolide is a labdane diterpenoid isolated from the stem and leaves of* Andrographis paniculata *(Chuanxinlian). Andrographolide, as a NF-*κ*B inhibitor, possesses anti-tumor activity and inhibits tumor angiogenesis. Andrographolide has been shown to reduce lesion size and improve in generalized hyperalgesia in a dose-dependent manner in an endometriosis rat model. This effect was mediated via COX-2, tissue factor (TF) and the phosphorylation of p50 and p65. Andrographolide dose could also suppress proliferation and cell cycle progression, reduce DNA-binding activity of NF-*κ*B, and inhibit COX-2 and TF expression in endometriotic stromal cells [128].

***Lithospermum erythrorhizon *(Zicao)**  is a Chinese herbal medicine with various antiviral and biological activities. Shikonin, an ingredient of* L. erythrorhizon*, has been shown to have wound healing, antimicrobial, anti-inflammatory, and antioxidant activities and is effective for several tumors; the underlying mechanism involves the inhibition of angiogenesis and has been observed both* in vivo *and* in vitro* [129]. Shikonin significantly inhibited the growth of human endometrial tissue implanted into severe combined immunodeficiency (SCID) mice via regulated upon activation normal T-cell expressed and secreted mRANTES levels in the peritoneal fluid; no adverse effects were observed during this process; shikonin significantly inhibited RANTES expression in U937 cells that were cultured either alone or co-cultured with human mesothelial and endometrial stromal cells* in vitro* [130].

In Chinese medicine, anti-angiogenic effects are mediated via multiple interdependent processes such as the modulation of gene expression, signaling pathways, and enzyme activities [131].

#### 3.4.1. Other Potential Anti-Angiogenic Chinese Herbs or Their Ingredients

Blood-activating and stasis-relieving drugs are widely used in Chinese medicine and are commonly applied in the treatment of various angiogenesis-related diseases. Several blood-activating and stasis-removing drugs have been associated with anti-angiogenic effects, although the regulatory mechanisms underlying these effects require further clarification in endometriosis. In the next section, we discuss the potential anti-angiogenic mechanisms of commonly used Chinese medicines.

***Ligusticum chuanxiong* (Chuanxiong)**  is an extremely common traditional, edible, medicinal herb.* L. chuanxiong* exerts therapeutic effects and has been applied in the treatment of both cardiovascular and cerebrovascular diseases. Approximately 174 compounds have been isolated and identified from* L. chuanxiong*; phthalides and alkaloids have been identified as the main bioactive ingredients with pharmacological properties [132, 133].

***Angelica sinensis *(Danggui)**  is one of the most commonly used drugs for gynecological diseases; n-butylidenephthalide (BP) has been identified as the bioactive component in a volatile oil of* Radix A. sinensis* (VOAS), and exerts angiogenic effects, both* in vitro* and* in vivo* [134].

***Heat-clearing and detoxifying drugs,*** such as other Chinese medicines, have also been associated with anti-angiogenic effects.*** Scutellaria baicalensis***(**Huangqin**) is a widely used for inflammation, diabetes, hypertension, cancer and virus related diseases. High dosage of baicalin or baicalein showed anti-angiogenesis effect through induction of apoptosis, but low dosage of baicalin promoted angiogenesis through increasing cell proliferation [135].*** Pulsatilla chinensis*** (**Baitouweng**)  extracts have anti-tumor activity against undifferentiated thyroid cancers via their apoptotic and anti-angiogenic effects [136]. The mechanism(s) underlying these anti-angiogenic Chinese herbs in endometriosis requires further investigation.

Potential anti-angiogenic natural compounds and Chinese herbs may provide anti-angiogenic drugs. For example, the following have all been associated with anti-angiogenic effects: Vitexins [137]; Luteolin [138];* Panaxatriol saponins* [139]; Actein [140];* Emodin* [141]; Paeonol;* Hedyotis diffusa* Willd and* M. tenacissima* are suggested to have anti-angiogenic effects [142].

In conclusion, several types of natural Chinese medicinal herbs are natural inhibitors of angiogenesis. It is therefore essential to understand their mechanistic basis for potential application in the treatment of endometriosis. For example, VEGF/VEGFR signaling plays a fundamental role in inducing vascular endothelial cell proliferation, migration, and angiogenesis. Many different types of Chinese medicine are also shown as natural inhibitors of angiogenesis. These also show a range of complexities. Collectively, these characteristics make it very difficult to carry out investigative research on such compounds. The the mechanism of these anti-angiogenic Chinese herbs in endometriosis needs further study. It is therefore essential to understand their mechanistic basis for potential application in the treatment of endometriosis.

### 3.5. Chinese Medicine Compounds

In traditional Chinese medicine, blood stasis syndrome (BSS) is an important pathology and is known to underlie many diseases [131]. The main endometriosis syndromes based on gynecology are as follows: qi stagnation with blood stasis; cold coagulation and blood stasis; kidney deficiency blood stasis; and qi deficiency blood stasis. Many Chinese herbs and formulas that promote blood circulation and the removal of blood stasis have definitive anti-platelet effects. These anti-angiogenic properties are widely used for the treatment of various diseases such as stroke, cardiovascular disease, and cancer [143–145].

In 1991, the Professional Committee of Obstetrics and Gynecology of the Chinese Association of Integrative Medicine published its integrative medicine standards for the diagnosis and treatment of endometriosis. The therapeutic plan for endometriosis was designed according to the age of the patient, her symptoms, the location and range of the lesion, and her requirement for fertility. The use of Chinese herbals in the treatment of endometriosis is based upon their anti-angiogenic effects. The use of traditional Chinese medicine includes the administration of pills, capsules, granules, and herbal decoctions. The following Chinese medicines had been reported to be effective for endometriosis treatment.** A list of Chinese medicine compounds used in the treatment of endometriosis treatment is provided in **Table 4.

***Guizhi Fuling Wan.***, a mixture of five herbal plants, is a classic formulation. Guizhi Fuling Wan, consists of* Cinnamomum cassia *(Guizhi),* Poria cocos *(Fuling),* Moutan Cortex *(Mudanpi),* Prunus persica *(Taoren), and* Paeonia lactiflora *(Chishao); these were identified in the classic monograph of traditional Chinese* medicine *“*Jingui Yaolue (Synopsis for the Golden Chamber, SGC)*” [146]. Guizhi Fuling Wan is widely used for the treatment of uterine fibroids, dysmenorrhea, endometriosis, and adenomyosis to improve blood stagnation in patients with a long history of disease. Twenty-seven potentially bioactive compounds including monoterpene glycosides, galloyl glucoses, acetophenones, phenylallyl compounds, and triterpenoids have been identified or tentatively characterized by high-performance liquid chromatography with tandem mass spectrometry (HPLC-DAD-MS / MS) [147].

Guizhi Fuling Wan has been shown to significantly inhibit the expression of proliferating cell nuclear antigen (PCNA) and platelet endothelial cell adhesion molecule-1(CD31) in the ectopic endometrium and significantly reduce the expression of VEGF and HIF-1*α* in the peritoneal fluid of rats model [148]. Other research has suggested that this Guizhi Fuling Wan may reduce of protein levels of HIF-1*α* and VEGF in ectopic endometrium and eutopic endometrium in endometriosis patients for one month treatment [149].

Futhermore, Guizhi Fuling Wan was shown to play an important role in the regression of endometriotic implants by immunological regulation in a rat model of endometriosis [150]. Guizhi Fuling Wan (capsule) also could inhibit mRNA levels of the apoptosis-inhibiting factor Bcl-2 by increasing mRNA levels for the apoptosis-promoting factor Bax. Guizhi Fuling Wan (capsule) could induce apoptosis in endometriotic cells and inhibit cell proliferation and metastasis of endometriotic cells via the mitochondrial apoptotic pathway [151]. Another study showed that Guizhi Fuling pills inhibit MEK and ERK pathways in patients with endometriosis [152]. Network pharmacology has demonstrated the effects of Guizhi Fuling Wan on uterine fibroids; the negative regulation of smooth muscle cell proliferation; apoptosis; and the Ras, wingless-type, epidermal growth factor and insulin-like growth factor-1 signaling pathways [153]. The effect of Guizhi Fuling Wan is mainly mediated via anti-angiogenesis and the promotion of cell apoptosis.

**Xiaochaihu Tang (XCHT)**  is also described in a “*Treatise on Cold Pathogenic and Miscellaneous Diseases*”, and comprised seven crude drugs:*Bupleurum chinense *(chaihu),* Scutellaria baicalensis *(Huangqin),* Ginseng root *(Renshen),* Pinellia ternata *(Banxia),* Glycyrrhiza uralensis *(Gancao),* Zingiber officinale *(Shengjiang), and* Ziziphus jujuba *(Hongzao). XCHT inhibited the growth and angiogenesis of the ectopic endometrium in a rat model. XCHT has been shown to reduce the level of VEGF and MVD in ectopic endometrium and decrease the level of IL-8 and TNF-*α* in blood and peritoneal fluid [154]. XCHT was able to decrease the expression of MMP-2 and MMP-9 in the ectopic endometrium in a rat model [155].

***Sanleng Wan***was first reported in the Chinese medical monograph “*Experiential effective prescription *(*Jingyan Liangfang*)” in Qing dynasty. This medicine is composed of traditional Chinese medicinal herbs found in* Sparganium stoloniferum* (Sanleng) and* Curcuma aeruginosa *(E'zhu) and is used for the treatment of dysmenorrhea. Sanleng Wan has been shown to reduce micro-vessel density (MVD) and inhibit VEGF, TNF-*α* protein, and mRNA levels in the ectopic endometrium in a rat model [156].

***Jiawei Sanleng Wan*** consists of* Sparganium stoloniferum *Buch. (Sanleng),* Curcuma phaeocaulis* (E'zhu), and* Astragalus membranaceus* (Huangqi). Jiawei Sanleng Wan has been shown to decrease the secretion of E2 by eutopic endometrial cells; an anti-angiogenic effect was obtained by reducing the level of VEGF in the eutopic endometrial cells during endometriosis [157, 158].

***Shaofu Zhuyu Decoction(SZD)*** originally recorded in the “*Correction of Errors in Medical Classics *(*Yiling Gaicuo*)”, a monograph compiled by Qingren Wang in the Qing dynasty. Shaofu Zhuyu Decoction (SZD) consists of ten commonly used herbs and has been widely used in clinical practice to relieve symptoms of endometriosis. SZD significantly reduced the size of ectopic lesions inhibited cell proliferation, increased cell apoptosis, and reduced MVD, HIF-1*α* and CD34 expression in the ectopic endometrium in a rat model [159].

***Sanjie Zhentong Capsules***,  a well-known traditionally prescribed Chinese medicine, consists of* Daemonorops draco draco *Bl. (Longxuejie),* Panax notoginseng *(Sanqi),* Fritillaria thunbergii *(Zhebeimu), and* Coix lacryma-jobi *(Yiyiren). These capsules, which are used for the treatment of endometriosis, effectively reduce endometriosis- and adenomyosis-associated pain, with few side effects [160]. Using ultra-high-performance liquid chromatography-tandem mass spectrometry (UHPLC-MS/MS), ten bioactive constituents were detected in rat plasma after the oral administration of Sanjie Zhentong capsules; these included peimine, peiminine, peimisine, loureirin A, loureirin B, 7,4'-dihydroxyflavone, pterostilbene, ginsenoside Rg1, ginsenoside Rb1, and notoginsenoside R1 [161]. Sanjie Zhentong capsules could effectively reduce the concentration of prostacyclin PGF2 and thromboxane B2 (TXB2) in plasma, thereby relieving uterine smooth muscle contraction, reducing inflammation, and enhancing immune function [162]. The two main components, oureirin and ginsenoside R, were shown to be effective in reducing the level of VEGF and TNF-*α* in the peritoneal fluids in an endometriosis rat model [163]. Sanjie Zhentong capsules and danazol both could inhibit ectopic lesions growth in a rat model of endometriosis, as well as reducing the levels of PGE2 in the peritoneal fluid [164].

***Eleng Capsules***, patented by the Guangdong Provincial Hospital of Chinese Medicine, have been used in the treatment of endometriosis for over 10 years now (National Patent: ZL2004 10033954.9). We conducted clinical and basic research on the Eleng capsule, which is composed of* Sparganium stoloniferum *(Sanleng),* Curcuma phaeocaulis *(E'zhu),* Slavia miltiorrhiza *(Danshen),* Curcuma wenyujin *(Yujin),* Trionyx sinensis *(Biejia),* Hirudo nipponica *(Shuizhi), and* Citrus aurantium *(Zhike). Long-term clinical and basic research confirmed that Eleng capsules are able to significantly and effectively relieve pain while also restoring fertility and inhibiting the recurrence of endometriosis by countering adhesion, invasion, and angiogenesis. Eleng capsules are also able to significantly relieve the symptoms and signs of endometriosis and blood stasis syndrome, potentially by down-regulating serum levels of prolactin (PRL), CA125, and anti-endometrial antibody (EMab) [165]. Eleng capsules also inhibited of angiogenesis by reducing the level of VEGF in eutopic and ectopic endometrium in endometriosis patients after four months treatment [166]. Other studies have suggested that Eleng capsules can regulate hemorheology, hormone levels, and immune function. Furthermore, Eleng capsules may also lower serum levels of soluble intercellular adhesion molecule-1(sICAM-1) and MMP-9 in patients with endometriosis, suggesting effects upon cell adhesion, invasion and angiogenesis [167].

***Dan***'***e Fukang Decoction***  is composed of* Slavia miltiorrhiza *(Danshen),* Curcuma phaeocaulis* (E'zhu),* Panax notoginseng* (Sanqi),* Paeonia lactiflora* (Chishao),* Angelica sinensis* (Danggui),* Sparganium stoloniferum* (Sanleng),* Cyperus rotundus* (Xiangfu),* Corydalis yanhusuo* (Yanhusuo),* Bupleurum chinense* (Chaihu), and* Glycyrrhiza uralensis* (Gancao). Dan'e Fukang decoction is known to reduce the expression of VEGF and TNF-*α* in the ectopic endometrium during endometriosis, while also inhibiting the invasion and proliferation of endometrial cells [168]; it also appeared to adjust the balance of MMP-9 and matrix metalloproteinase inhibitor-1 (TIMP-1) in endometriosis rat model [169]. Dan'e Fukang decoction may also be associated with the reduced expression of IL-17 and IL-23 in the ectopic endometrium of rats model [170].

#### 3.5.1. Other Chinese Herbal Decoctions and Compounds

Many traditional Chinese medicine decoctions have been shown to have anti-angiogenic effects. Bushen Wenyang Huayu decoction reduced levels of VEGF and CA125 in the serum of endometriosis patients [171]. Xiaozheng decoction also could relieve dysmenorrhea and pelvic pain and improve qi stagnation and blood removal in endometriosis patients, which may be related to lower levels of CA125, CA199, MMP-3, MMP-9, VEGF, and TNF-*α* in serum [172]. Clearing heat and expelling blood stasis treatment reduced the level of angiogenesis and inflammatory-related factors in serum and peritoneal fluid in a rat endometriosis model by inhibiting the expression of VEGFR2 and COX-2 in the ectopic endometrium. These methods influenced angiogenesis and peritoneal inflammation in the local microenvironment by inhibiting growth of ectopic endometrium [173]. Taoren Yujin decoction significantly reduced the serum levels of E2, CA125, and EMAb, as well as the expression of MVD, VEGF, and Ang-2 in a rat model [174]. Huayu Xiaozheng decoction could inhibit angiogenesis and reduce endometriotic implant volume and histopathological scores through a reduction in VEGF and angiopoietin-2(Ang-2) expression in the ectopic endometrium in a rat model [175]. Wenshen Xiaozheng Tang treatment significantly decreased the lesion size and inhibited cell proliferation in the endometriotic lesions in a rat model by reducing the expression of HIF-1*α* in the endometriotic lesions and decreasing concentration of VEGF in peritoneal fluid [176].

Herbal extracts and Chinese medicines are therefore of significant value for the treatment of endometriosis, largely due to their anti-angiogenic properties and their ability to directly affect cell function. Furthermore, the individualized use of herbal treatments is designed to meet the needs of each patient.

## 4. Discussion

### 4.1. Traditional Surgery and Drug Treatment: Problems and Challenges

Endometriosis is a complex gynecological disease. Endometriosis is not a potentially life-threatening disease, and most of the affected patients are young women of reproductive age. Current medical treatments mainly address the patient's symptoms such as pain, fertility, and recurrence. However, emerging therapeutic approaches, including GnRH antagonists, aromatase inhibitors, anti-angiogenic drugs, or alternative medicines, provide new tools for the treatment of endometriosis [2, 9]. Most of the affected women are of reproductive age and are not able to endure the adverse reactions associated with some current treatments, such as perimenopausal syndrome and osteoporosis. Surgery can relieve the pain experienced by patients, but the risk is high. Therefore, there is a need to investigate other therapies with higher efficacy, fewer side effects, and a lower recurrence rate. However, current research on endometriosis is still insufficient.

In particular, research into the treatment of endometriosis needs to address the protection of ovarian reserve, the prevention of recurrence, and the reduction of side effects and to seek to develop long-term treatment options. From this perspective, anti-angiogenic agents are particularly promising because they have favorable safety profiles and could act as antiangiogenic treatments aimed at inhibiting new vessel formation. Targeting angiogenesis could be a suitable method with which to prevent recurrent endometriosis conditions associated with current pharmacological and surgical treatment modalities. Understanding the roles of angiogenesis in the pathophysiology of endometriosis is essential for the development of novel therapeutic approaches.

However, existing information relating to the efficacy of exclusive and most inclusive anti-angiogenic compounds has been obtained from animal models [57]. Another major challenge for anti-angiogenic therapy is the selective targeting of pathological angiogenesis without altering the physiological angiogenesis necessary for reproductive function or wound healing [8, 177, 178]. Anti-angiogenic therapy may alter reproductive function by damaging physiological angiogenesis, the most worrying risk being the increased potential for teratogenicity.

Alternative medicines for the treatment of endometriosis are widely used in China, where these treatments improve patient symptoms effectively, although large-scale studies have not yet been completed. Natural compounds, along with complementary and alternative medicines, are significant because of their multi-target characteristics, which provide new sources for difficult and chronic diseases. The mechanistic basis for the anti-angiogenic effect of many natural compounds and traditional Chinese medicines, along with complementary and alternative medicines, is still unclear. There are still many compounds and treatments that are unidentified and worthy of further exploration [179].

### 4.2. The Advantages of Alternative and Complementary Medicine in the Treatment of Endometriosis

**Figure 1:**
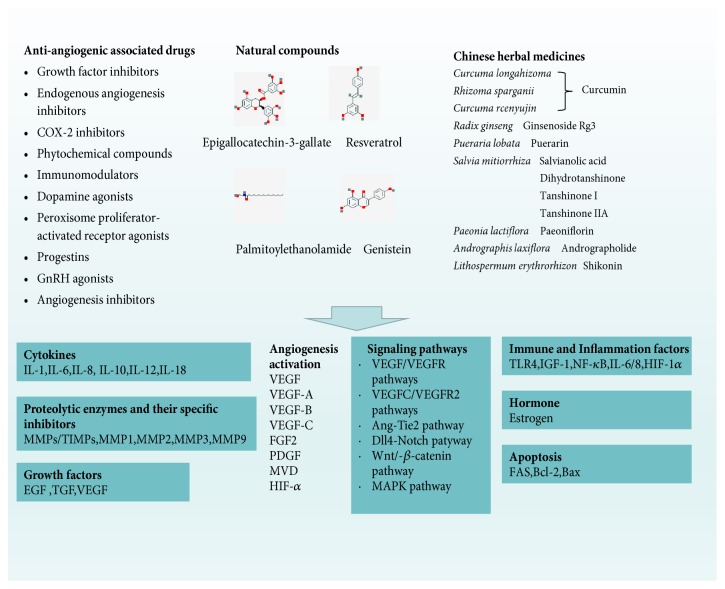
The mechanisms underlying the action of natural compounds and Chinese herbs.

Alternative and complementary medical treatments primarily consist of natural compounds and herbals, which have fewer side effects and are suitable for long-term treatment and are acceptable to patients. Supplementary alternative medicine is widely used worldwide, particularly in Asian countries. Targeting natural compounds may provide a new direction for finding new targets for endometriosis treatment. Traditional Chinese medicine treatment is based on compounds with multi-target regulation characteristics and can intervene in the disease process via multiple pathways. In terms of angiogenesis, natural medicines and traditional Chinese medicines are involved in the regulation of multiple pathways and are associated with pathways such as inflammation and immunity to jointly regulate the process of angiogenesis. Natural compounds and traditional Chinese medicine for the treatment of endometriosis have multi-channel and multi-target regulatory characteristics, which are mainly related to the down-regulation of VEGF.** The mechanisms underlying the action of natural compounds and Chinese herbs are summarized in **Figure 1.

Chinese medicine treatments have been used for the symptoms of endometriosis for more than two thousand years. These treatments have been demonstrated to be beneficial in relieving dysmenorrhea, in regulating menstruation to assist pregnancy, and in reducing pelvic mass. According to the theory of Chinese medicine, endometriosis is caused by blood stasis. Blood stasis forms a mass in the body, which causes pain, dysmenorrhea, and infertility [179]. As reported in literature, the TCM treatment of endometriosis has no obvious side effects and can be taken in pregnancy.* Practice Guidelines for the Treatment of Endometriosis with Chinese Herbal Medicine* were developed using the Delphi Process in 2007 [180]. Guidelines in 2015 for the diagnosis and treatment of endometriosis in China, issued by the* Endometriosis Collaborative Group of the Chinese Medical Association Obstetrics and Gynecology Branch*, mentioned dysmenorrhea also can consider Chinese medicine treatment [181]. Most Chinese medicine prescriptions feature multiple substances, which affect a range of biological pathways and targets. The precise therapeutic mechanisms underlying the effects of these Chinese medicines have not yet been clarified but may involve multiple effects such as the regulation of endometriosis invasion, adhesion, and angiogenesis.

In summary, our literature review indicates that Chinese medicine mainly regulates angiogenesis-related factors and angiogenesis pathways, reduces angiogenesis, and reduces the density of microvessels. VEGF/VEGFR signaling pathways and related signaling pathways represent specific foci for current research. Furthermore, Chinese medicine may play a synergistic role with other regulatory mechanisms.

**Figure 2:**
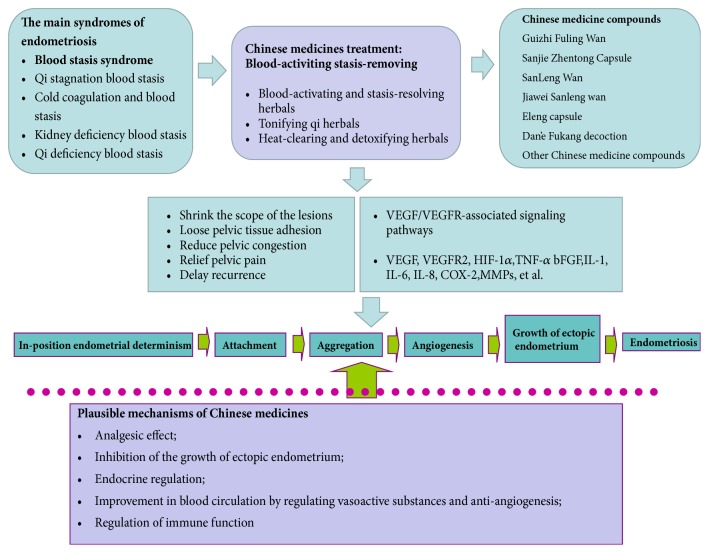
Therapeutic mechanisms of Chinese medicines in the treatment of endometriosis-associated angiogenesis.

***There are five other mechanisms that should be considered***.  First, the analgesic effect: clinical studies suggest that Chinese herbals can alleviate the pain symptoms of patients with endometriosis, and that this might be related to the regulation of prostaglandin-related receptors [167, 171]; second, inhibition of the growth of ectopic endometrium [152, 170]; third, endocrine regulation: endometriosis is an estrogen-dependent disease and estrogen, a periodically secreted hormone, regulates the proliferation, secretion, and shedding of ectopic endometrium. Natural medicine and Chinese medicine are able to regulate estrogen [120]; fourth, improvements in blood circulation via the regulation of vasoactive substances and vascular-related factors in order to relieve pain; fifth, the regulation of immune function: immunological dysfunction has been proposed as a critical facilitator of ectopic lesion growth following retrograde menstruation [25]. Chinese medicine could potentially regulate immune responses in endometriosis patients [179]. There may also be synergies between various mechanisms.** See **Figure 2** for a summary of Chinese medicine compounds that show complex regulatory mechanisms. **

### 4.3. The Lack of Research into the Use of Alternative and Complementary Medicine for the Treatment of Endometriosis

At present, studies on the anti-angiogenesis activities of monomer and Chinese herbal extracts are very transparent; however, basic research on compounds remains in its infancy. In the theory of TCM, formulae with multiple combinations of herbs are more frequently used for clinical therapy [182]. The complexity of alternative and complementary medicines makes it extremely difficult to systematically explain molecular mechanisms adequately using routine methodology [183]. Therefore, studies of Chinese medicines should be comparatively enhanced to explore more alternative and complementary medicines with potent therapeutic effects.

The bioavailability of these compounds is low because many of these molecules have poor solubility; therefore, only negligible concentrations of these natural compounds can reach the peripheral circulation and the desired sites affected by disease. We also need to identify the biological target(s), disease pathway(s), and the active compound(s) that are responsible for the modification of endometriosis. Furthermore, there is a lack of confirmatory therapeutic studies that establish the molecular mechanistic basis for therapies; this requires further investigation and confirmation.

From retrospective TCM compound clinical research, we can see that there is a clear lack of literature relating to multi-channel and signaling pathway mechanisms. Notably, the mechanisms underlying the anti-angiogenic therapeutic activities of traditional Chinese medicine still remain unclear. Furthermore, the clinical practice of TCMs has not yet been reviewed and examined in the context of modern medical knowledge, and there is a clear lack of lack of large, randomized, and controlled trials. For example, one meta-analysis study was shown that Chinese Medicine Bushen Huoxue Prescription (BSHXP) significantly improved endometriosis-related symptoms and syndromes. But the evidence is inconclusive because of the low methodological quality of the included RCTs [184]. Clinical practice has not yet been reviewed and examined in the context of modern medical knowledge, and there is a notable absence of large, randomized, and controlled trials [12].

It is also poor trial outcomes and the lack of transparency in endometriosis, as well as in alternative and complementary medicines in endometriosis treatment. Several completed clinical trials on endometriosis did not publish their results, and presumably failed. Elucidating the causes for failed clinical trials may improve future researches [185].

### 4.4. New Methods to Clarify the Mechanistic Basis of the Effects of Natural Compounds and Chinese Herbs

Natural compounds and Chinese herbs are capable of regulating multiple targets, with potential applications in complex diseases. New methods must be adopted to investigate Chinese compounds. Network pharmacology can predict the target profiles and pharmacological actions of herbal compounds, reveal drug-gene-disease comodule associations, and thus allow us to screen synergistic multi-compounds from herbal formulas using high-throughput evaluation. Network pharmacology and systems pharmacology should therefore be used more effectively to identify herbal ingredients and their related properties to establish compound-target relationships and target-disease relationships [186–188].

Genome-wide association studies have demonstrated several reproducible loci associated with endometriosis. Such studies could identify informative subtypes of endometriosis that will enhance our understanding of the pathogenic mechanisms of endometriosis [189]. It is particularly important to clarify targets for the regulation of angiogenesis in endometriosis. Treatments that are targeted to genes associated with endometriosis remain a significant future objective. Multiple approaches, including pharmaco-genomics, -proteomics, -transcriptomics, and -metabolomics, are rapidly being developed to study the pharmacology of Chinese medicine [190, 191].

Furthermore, clinical endometriosis research, featuring high-quality Chinese herbal compounds, needs to focus on evidence-based medicine. The phenotypes of patients with endometriosis are different, and the further application of precision medical methods, combined with the use of complementary or alternative medicines, is expected to provide personalized forms of treatment for patients with endometriosis [1]. Future research, with a larger numbers of participants, is required to substantiate exploratory results and to establish the role of Chinese medicine as a standalone medical option or as a post-surgical adjunct for treatment of endometriosis. Real world studies (RWS) have become a significant hotspot for clinical research [192, 193]. Big data in real-world clinical research could be used in the complementary alternative medical treatment of endometriosis to obtain more clinical evidence, which is more in line with the research needs of chronic disease. Natural and Chinese medicines have been associated with reports of adverse reactions. For the long-term use of natural compounds and Chinese medicines, it is necessary to further clarify safety profiles in both clinical and basic experiments. Otherwise, we need to avoid and minimize such side effects. Nevertheless, the prescription of Chinese herbals should follow strict diagnosis and treatment principles.

## 5. Conclusion

Endometriosis is both a gynecological and chronic disease. Angiogenesis is a primary mechanism involved in endometriosis. In experimental studies, various anti-angiogenic agents have induced the regression of endometriotic lesions by targeting their blood supply. Angiogenesis inhibitors may therefore become new targets for the treatment of endometriosis [33, 194]. Most patients with endometriosis are women of childbearing age and need to consider their fertility requirements and long-term treatment safety. However, angiogenesis inhibitors have side effects and may not be suitable for the treatment of patients with endometriosis [66]. We urgently need to further clarify the pathogenesis of endometriosis and explore options for safe and effective therapies. In this review, we found that anti-angiogenic alternative and complementary medicines have multi-channel and multi-target regulatory characteristics. Natural products and Chinese herbs exert anti-angiogenic activity via the direct and indirect targeting of angiogenesis signaling pathways, and by inhibiting the VEGF/VEGFR pathway. These are the most common and likely mechanisms of action for angiogenesis inhibitors and may represent a potential source of drugs for the treatment of endometriosis. Traditional Chinese medicine has a complex composition, and its anti-angiogenic effects may be related to its pharmaceutically active components. Compared with currently available anti-angiogenic drugs, natural products not only may have similar therapeutic potential but are also inexpensive, less toxic, and easy to administer. Agents derived from natural compounds and Chinese medicines have shown their multitarget curative potential by impairing angiogenic stimulatory signaling pathways directly or eliciting synergistically therapeutic effects with anti-angiogenic drugs. We believe that natural medicine will be the main direction for future drug development in endometriosis treatment. Nevertheless, we also recognize that existing investigations of medicinal plants and their therapeutic use are very limited. In many cases, these studies were only based on* in vitro* research or animal models. Furthermore, there is not enough convincing clinical evidence for the potential use of these herbs for the treatment of endometriosis. In the era of evidence-based precision medicine, more clinical trials are now necessary to prove the efficacy and safety of these therapies in patients with endometriosis, as well as to explore the mechanistic basis of their therapeutic effect. Future research should also be performed to establish pharmacological evidence for phytochemical effects using pharmaco-genomics, -proteomics, -transcriptomics, and -metabolomics. Studies should also investigate the efficacy and effectiveness of the herbal combinations and therapeutic strategies used in current herbal practice.

## Figures and Tables

**Table 1:** Common anti-angiogenic medications used for the management of endometriosis.

**Compound/Drugs**	**Categories**	**Molecular mechanisms**	**Major targets**		**Type**	**References**
			**Anti-angiogenesis targets**	**Others associated targets**		
Degarelix, Gonadorelin, Triptorelin/ GnRH antagonists	Endocrine therapy Gonadotropin-releasing hormone antagonist Hormone antagonists and related agent	Regression of the endometriotic lesions Increased the apoptotic rate Anti-angiogenesis	VEGF-A↓ TNF-a ↓	Bax↑ FasL↑ Bcl-2↓ IL-1↓ IL-8 ↓	Endometriotic stromal cells	[35]

Dienogest	Contraceptive agents Hormone antagonist Cytochrome P-450 CYP3A4 substrate	Reduced proliferation Increased apoptosis	MVD↓	Ki67↓	In human endometriosis cells	[36]

Bevacizumab	Angiogenesis inhibitor	Inhibited cell proliferation Reduced vascular density Increased apoptosis	VEGF↓		BALB/c mouse model	[37–39]

Ranibizumab	Angiogenesis inhibitor	Reduced endometriotic volumes	VEGF ↓		Rat model	[40]

Thalidomide	Angiogenesis inhibitor	Reduced endometriotic volumes Enhance apoptosis	VEGF-A ↓ myeloperoxidase ↓		Rat model	[41, 42]

Sunitinib	Angiogenesis inhibitor	Enhanced apoptosis	VEGF-A ↓	CD117 ↓	Rat model	[43–45]

Lovastatin Statins	Lipid-lowering agent Hypolipidemic agent	Inhibited angiogenesis and cell proliferation Increased the differentiation of eMSCs Decreased expression of eMSCs markers		BMP2↑ cyclo-oxygenase (COX), and hypoxia-inducible factor 1*α* (HIF-1)RUNX2 ↑	Endometrial mesenchymal stem cells (eMSCs)	[46]

Simvastatin Statins	Lipid-lowering agent Cytochrome P-450 enzyme inducer/inhibitor	Inhibited the proliferation and the contractility Decreased endometrial implants	VEGF↓ HIF-1*α*↓	MMP-3 ↓	Human endometriotic stromal cells/clinical research/primate Model	[47–51]

Cabergoline Dopamine agonist	Cytochrome P-450 enzyme inhibitors Dopamine agonists Ergot-derivative dopamine receptor agonists Serotonin 5-HT1 receptor agonists	Inhibited the growth of established endometriosis lesions	VEGFR-2 ↓		Clinical research/rat and mouse models	[52–54]

Quinagolide Dopamine agonist	Dopamine agonist non-ergot-derived selective dopamine D2 receptor agonist	Reduced the levels of IL-6 and VEGF in peritoneal fluid	VEGF ↓	IL-6 ↓	Rat model	[55]

Endostatin Endostar	Angiogenesis inhibitor	Suppression of VEGF in peritoneal fluid	VEGF ↓ MVD↓	MMP-2↓	Mouse and rat models	[56]

Angiostatin	Angiogenesis inhibitor	Reduction of estradiol and progesterone production.		E2↓	Estrogen-supplemented ovariectomized mouse model	[57]

Rosiglitazone	Antidiabetic drug Thiazolidinedione	Inhibited aromatase and COX-2 expression Reduced the vascularized area of lesions Enhanced apoptosis	CD31 ↓ CD34 ↓	PGE2 ↓ PCNA ↓	Human cell culture/ BALB/C mice model	[29]

Fenofibrate Statins	Lipid-lowering agent Cytochrome P-450 enzyme inducer/inhibitor	Reduction of endometriotic lesion	VEGF ↓		Rat model	[59]

HIF-1*α*: hypoxia-inducible factor 1*α*; IL-1: interleukin-1; MPO: myeloperoxidase; MVD: microvessel density; VEGF: vascular endothelial growth factor.

**Table 2:** Natural product inhibitors of angiogenesis for endometriosis.

**Natural compounds**	**Molecular targets/mechanisms**		**Major targets**		**Type**	**Reference**
	**Anti-angiogenesis mechanisms**	**Others mechanisms**	**Anti-angiogenesis targets**	**Others associated Targets**		
Epigallocatechin gallate (EGCG)/Pro-Epigallocatechin gallate (pro-EGCG)	Inhibited VEGFC/VEGFR2 signaling pathways	Reduced lesion size Inhibited cellular oxidation Prevented free radical damage to cells Enhanced lesion apoptosis Inhibited MAPK/Smad signaling pathways	VEGFR2 ↓ VEGF-C↓	NF-*κ*B ↓ E2 ↓ TGF-*β*1 ↓	Endometrial and endometriotic stromal cells/ Mouse model	[71–76]

Resveratrol	Reduced micro-vessel density (MVD)	Anti-oxidative properties Anti-inflammatory Enhanced apoptosis in endometriotic Stromal Cells Inhibited vascularization and cell proliferation	VEGF↓ TNF-*ɑ*↓	MCP-1 ↓ MMP-2 ↓ MMP-9 ↓ IL-6 ↓ IL-8 ↓ HMGCR ↓ ESR1↓ CA125 ↓	Endometriotic stromal cells (ESCs)/Rat model/Clinical trials	[77–85]

Palmitoylethanolamide	Reduced VEGF	Inhibited development of endometriotic lesions Reduced peroxynitrite formation	VEGF↓ NGF↓	VCAM-1↓ MMP-9↓ poly-ADP↓ Ik-B*ɑ*↓ NF-*κ*B↓	Rat model	[86]

*Achillea biebersteinii* Afan	Reduced peritoneal fluid levels of TNF-*α*, VEGF	Reduced endometriotic volumes	VEGF↓ TNF-*ɑ*↓	IL-6↓	Sprague Dawley rats model	[87]

*Viburnum opulus L.*	Reduced VEGF	Reduced endometriotic volumes	VEGF↓ TNF-*ɑ*↓,	IL-6↓	Rat model	[88]

Genistein (Flavonoids)	Regulated angiogenesis	Regulated inflammation Reduced the expression of estrogen receptor *α* Increased the expression of estrogen receptor *β*	VEGF↓	HIF-1*α*↓	Murine model	[91, 92]

Xanthohumol (prenylated flavonoid)	Suppressed vascularization in endometriotic lesions	Inhibited the development of endometriotic lesions Anti-proliferative Anti-inflammatory	MVD ↓	Phosphoinositide 3-kinase protein↓	BALB/c mouse model	[93]

*Sea buckthorn and St. John's *wort	Reduced the level VEGF in peritoneal fluids	Inhibited the development of endometriotic lesions	TNF-*α*↓ VEGF↓	IL-6↓	Rat model	[94]

Naringenin (Flavonoids)	Regulated VEGF/KDR signaling pathway in human endothelial cells	Inhibited proliferation Increased apoptosis Regulated MAPK and AKT signal transduction pathways in human endometriosis cell lines	VEGF↓ KDR(VEGFR2) ↓	PI3K↓ MAPK↓	In (VK2/E6E7 and End1/E6E7)human endometriosis cell lines/ Human endothelial cells	[95, 96]

CA125: carcinoembryonic antigen; ESR1: estrogen receptor-*ɑ*; HMGCR: 3-hydroxy-3-methylglutaryl-CoA reductase; IL-6: interleukin (IL)-6; MAPK: mitogen-activated protein kinase; MCP-1: monocyte chemoattractant protein-1; MMP-2: matrix metalloproteinases-2; MMP-9: matrix metalloproteinases-9; MVD: microvessel density; NGF: nerve growth factor; poly-ADP: lymphocyte accumulation and reduced peroxynitrite formation; TGF-*β*1: transforming growth factor; TNF-*α*: tumor necrosis factor-*α*.

**Table 3:** Main anti-angiogenesis agents for endometriosis that have been isolated from Chinese herbal medicines.

**Chinese herbal ** ** medicines ** Latin name/Chinese name	**The main chemical compounds**	**Molecular** ** targets/** **mechanisms**	**Preclinical or clinical evidence of ant-angiogenesis**	**Major Targets**		**Signal pathway**	**Type**	**reference**
				**Anti-angiogenesis targets**	**Others associtated targets**			
*Curcuma longa *(Jianghuang) *Rhizoma curcumae*(E'zhu) *Rhizoma sparganii* (Sanleng) *Curcuma rcenyujin* (Yujing)	Curcumin	Inhibited angiogenesis, Induced cell apoptosis	Anti-inflammatory, antioxidant, anti-tumor, anti-angiogenesis, anti-metastatic activities Cell proliferation and apoptosis	VEGF↓ MVD↓ FGF↓ PDGF↓ TNF-*α*↓	MMP-3↓ MMP-9↓ NF-*κ*B↓ p53 ↓ E2↓ PGE2↓ CA-125↓ G1-phase↓ EGF↓ HER-2↓ IGF-1↓	VEGF signaling pathway	Rat model/In humans	[103–109]

*Radix ginseng* (Renshen)	Ginsenoside Rg3	Induced cell apoptosis, Inhibited angiogenesis	Immune enhancement Antioxidant Anti-inflammatory Anti-neuroprotective Anti-metabolic syndrome	VEGF↓ VEGFR-2↓ ↓	miRNA-27b-3p ↓	PI3K/Akt/mTOR signaling pathway	Endothelial cells/Rat model/human embryonic stem cells (HESCs)	[110–114]

*Pueraria lobata* (Gegen)	Puerarin	Induced cell apoptosis, Inhibited angiogenesis	Estrogen receptors, Proliferation of endometriotic stromal cells (ESCs)	VEGFA ↓ Ang-1 ↓ Ang-2 ↓	E2↓ PGE2↓ p450 ↓ Cox-2 ↓ cyclin D1↓ cdc25A↓	VEGF signaling pathway	Endometriotic stromal cells	[115–120]

*Salvia miltiorrhiza* (Danshen)	Tanshinone II A (other compounds: salvianolic acid, dihydrotanshinone, tanshinone I,et al)	Inhibited angiogenesis	Reduced cell viability Induced apoptosis Inhibited cell migration and invasion Reduced the expression of 14-3-3*ζ* in EESCs	VEGFR2 ↓ TNF-a↓	p53↓ p21 ↓ DMD2↓ sICAM-1↓ sVCAM-1 ↓ CA125↓ MMP-9↓ IL-18↓ IL-13↓	VEGFR2 pathway, Akt/JNK signaling pathways	Ectopic endometrial stromal cells	[121–126]

*Paeonia lactiflora *(Chishao)	Paeoniflorin	Inhibited angiogenesis	Anti-inflammatory Rrelieved of pain Influenced estrogen receptor *α* (ESR*α*)	HIF-1*α*↓	ESR*α*↓		In Human/At network pharmacological and pharmacodynamic levels	[127]

*Andrographis paniculata* (Chuanxinlian)	Andrographolide	Inhibited tumor angiogenesis	Reduced lesion size in a rat model Improved in generalized hyperalgesia Inhibited COX-2 and TF expression Influenced phosphorylated p50 and p65 Suppressed proliferation and cell cycle progression Attenuated DNA-binding activity of NF-*κ*B	NGF↓	NF-*κ*B↓ COX-2↓ TF↓		In endometriotic stromal cells/Rat mode	[128]

*Lithospermum erythrorhizon* (Zicao)	Shikonin	Inhibited angiogenesis	Inhibited the growth of human endometrial tissueInhibited the chemotaxis of monocytes,regulated normal T-cell expressed and secreted (mRANTES) levels in a murine model Inhibited RANTES expression				In a murine model of endometriosis/In U937 cells	[129, 130]

Ang-1: Angiopoietin-1; Ang-2: Angiopoietin-2; Akt: protein kinase B; JNK” c-Jun N-terminal kinase; MDM2: murine double minute2; NF-*κ*B: nuclear factor-kappa B; p450arom: cytochrome P450; sICAM-1: soluble intercellular adhesion molecule-1; sVCAM-1: soluble vascular cell adhesion molecule-1; TF: tissue factor.

**Table 4:** Main anti-angiogeneic compounds for endometriosis that have been isolated from herbs.

**Compounds Name**	**Component/herb** **Latin name/Chinese name**	**Mechanisms**	**Factors**		**Type**	**reference**
			**Angiogenesis targets**	**Other associated Targets**		
Guizhi Fuling Wan	*Cinnamomum cassia *(Guizhi) *Poria cocos *(Fuling) *Moutan Cortex *(Mudanpi) *Prunus persica *(Taoren) *Paeonia lactiflora *(Chishao)	Inhibited the proliferation of endometrial cells and cell division cycle Promoted cell apoptosis	CD31↓ VEGF↓ HIF-1*α*↓	PCNA↓ Bcl-2↓ Bax↑ MEK↓ ERK↓	Rat model/In endometriosis patients	[146–153]

Xiaochaihu Tang	Bupleurum chinense (Chaihu) *Scutellaria baicalensis* (Huangqin) *ginseng root *(Renshen) *Pinellia ternata *(Banxia) *Glycyrrhiza uralensis *(Gancao) *Zingiber officinale *(Shengjiang) *Ziziphus jujuba *(Hongzao)	Inhibited the growth and angiogenesis	VEGF↓ MVD ↓	IL-8↓ TNF-*α*↓ MMP-2↓ MMP-9↓	Rat model	[154, 155]

Sanleng Wan	*Sparganium stoloniferum Buch.* (Sanleng) *Curcuma aeruginosa Roxb. * (E'zhu)	Anti-angiogenesis	MVD ↓ VEGF↓ TNF-*α*↓	E2↓	Rat model	[156]

Jiawei Sanleng Wan	*Sparganium stoloniferum Buch. *(Sanleng) *Curcuma phaeocaulis *(E'zhu) *Astragalus membranaceus* (Huangqi)	Anti-angiogenesis, Inhibited the eutopic endometrium cells	VEGF↓	E2↓	Rat model/the eutopic endometrial cells	[157, 158]

Shaofu Zhuyu Decoction	*Foeniculi Fructus *(Xiaohuixiang) *Zingiberis Rhizoma *(Ganjiang) *Corydalis Rhizoma *(Yanhusuo) *Commiphora myrrha *(Moyao) *Ligusticum chuanxiong* (Chuanxiong) *Angelica sinensis *(Danggui) *Paeonia lactiflora *(Chishao) *Cinnamomum cassia *(Rougui) *Typha angustifolia *(Puhuang) *Trogopterus xanthipes *(Wulingzhi)	Reduced the size of ectopic lesions Inhibited cell proliferation Increased cell apoptosis	MVD ↓ HIF-1*α* ↓ CD34 ↓		Rat model	[159]

Sanjie Zhentong Capsules	*Daemonorops draco Bl. *(Longxuejie) *Panax notoginseng * (Sanqi) *Fritillaria thunbergii Miq. * (Zhebeimu) *Coix lacryma-jobi L.* (Yiyiren)	Reduced endometriosis-associated pain Anti-inflammatory Enhanced immune function	VEGF↓ TNF-*α*↓	PGF2↓ TXB2↓	Rat model	[160–164]

Eleng capsules	*Sparganium stoloniferum *(Sanleng) *Curcuma phaeocaulis *(E'zhu) *Slavia miltiorrhiza *(Danshen) *Curcuma wenyujin *(Yujin) *Trionyx sinensis *(Biejia) *Hirudo nipponica *(Shuizhi) *Citrus aurantium *(Zhike)	Inhibited the growth of ectopic endometrium Anti-adhesion, invasion and angiogenesis	VEGF↓	PRL↓ CA125↓ EMAb↓ sICAM-1↓ MMP-9↓	Rat model	[165–167]

Dan'e Fukang decoction	*Slavia miltiorrhiza * (Danshen) *Curcuma phaeocaulis *(E'zhu) *Panax notoginseng *(Sanqi) *Paeonia lactiflora Pall. *(Chishao) *Angelica sinensis *(Danggui) *Sparganium stoloniferum *(Sanleng) *Cyperus rotundus *(Xiangfu) *Corydalis yanhusuo *(Yanhusuo) *Bupleurum chinense *(Chaihu) *Glycyrrhiza uralensis *(Gancao).	Inhibited the invasion and proliferation of endometrial cells	VEGF↓ TNF-*α*↓	MMP-9↓ TIMP-1↓ IL-17 ↓ IL-23↓	Rat model	[168–170]

Bushen Wenyang Huayu Decoction	*Aconitum carmichaeli* (Fuzi) *Cinnamomum cassia *(Rougui) *Foeniculum uulgare *(Xiaohuixiang) *Corydalis yanhusuo *(Yanhusuo) *Commiphora myrrha *(Moyao)	Relieved chronic pelvic pain and dysmenorrhea	VEGF ↓	CA125 ↓	In endometriosis patients	[171]

Xiaozheng decoction	*Astragalus membranaceus *(Huangqi) *Coix lacryma-jobi* (Yiyiren) *Patrinia villosa* (Baijiangcao) *Salviae miltiorrhizae* (Danshen) *Poria cocos *(Fuling) *Paeonia suffruticosa *(Mudanpi) *Paeonia lactiflora *(Chishao) *Cyperus rotundus *(Xiangfu) *Corydalis repens *(Yanhusuo) *Eupolyphaga* (Tubiechong) *Liquidambar *(Lulutong) Gleditsia sinensis (Zaojiaoci) Lindera aggregata (Wuyao) Cyathula officinalis (Chuanniuxi) *Cinnamomum cassia *(Guizhi) Dipsacus asper (Xuduan)	Relieved dysmenorrhea Improved qi stagnation and blood removal	VEGF↓ TNF-*α*↓	CA125↓ CA199↓ MMP-3↓ MMP-9↓	In endometriosis patients	[172]

Clearing heat and expelling blood stasis method	*Polygonum emodi *(Hongteng) *Typha angustifolia *(Shengpuhuang) *Concha Ostreae *(Muli) *Corydalis repens *(Yanhusuo) *Semen Persicae *(Taoren) *Paeonia suffruticosa *(Mudanpi) *Cyperus rotundus *(Xiangfu)	Educed the level of angiogenesis Anti-inflammatory	VEGFR2↓	COX-2↓	Rat model	[173]

Taoren Yujin Decoction	*Semen Persicae *(Taoren) *Curcuma wenyujin *(Yujin)	Reduced microvascular density Anti-angiogenesis	MVD↓ VEGF↓ Ang-2 ↓	E2↓ CA125↓ EMAb↓	Rat model	[174]

Huayu Xiaozheng Decoction	*Salviae miltiorrhizae* (Danshen) *Morinda officinalis* (Bajitian) *Panax notoginseng* (Sanqi) *Coix lacryma-job*i (Yiyiren) *Fritillaria thunbergii* (Zhebeimu) *Prunella vulgaris* (Xiakucao) *Polygonum aviculare* (Bianxu) *Rheum palmatum* (Dahuang) Dianthus superbus (Qumai) Corydalis yanhusuo (Yanhusuo) Whitmania pigra Whitman (Shuizhi) Typha angustifolia (Puhuang) Daemonorops draco (Xuejie)	Anti-angiogenesis	VEGF↓ Ang-2 ↓		Rat model	[175]

Wenshen XiaozhengTang	*Typha angustifolia *(Puhuang) *Trogopterus xanthipes *(Wulingzhi) *Draconis Sanguis *(Xuejie) *Astragalus membranaceus * (Huangqi) * Dipsacus asper *(Xuduan) *Commiphora myrrha *(Moyao) *Corydalis Rhizoma *(Yanhusuo) *MeLia toosendan *(Chuanlianzi)	Decreased the lesion size Inhibited cell proliferation	HIF-1*α* ↓ VEGF ↓		Rat model	[176]

CD31: platelet endothelial cell adhesion molecule-1; EMab: anti-endometrial antibody; PCNA: proliferating cell nuclear antigen; PRL: prolactin; TIMP-1: matrix metalloproteinase inhibitor-1; TXB2: thromboxane B2.

## Abbreviation

Ang-1: Angiopoietin-1
Ang: Angiotensin
COC: Combined oral contraceptive
COX: Cyclo-oxygenase
HIF-1: Hypoxia-inducible factor 1*α*
MCP-1: Monocyte chemoattractant protein-1
EMSCs: Endometrial mesenchymal stem cells
ESCs: Endometriotic stromal cells
EESCs: Ectopic endometrial stromal cells
ESR1: Estrogen receptor-*ɑ*
MMPs: Matrix metalloproteinases
MVD: Microvessel density
MDM2: Murine double minute2
NF-*κ*B: Nuclear factor-kappa B
NSAIDs: Nonsteroidal anti-inflammatory drugs
sICAM-1: Soluble intercellular adhesion molecule-1
sVCAM-1: Soluble vascular cell adhesionmolecula-1
NGF: Nerve growth factor
PGE2: Prostaglandin E2
TF: Tissue factor
TGF: Transforming growth factor
TIMP: Matrix metalloproteinase inhibitor
TLR4: Toll like receptor-4
TNF-*ɑ*: Anti-tumor necrosis factor-*ɑ*
VEGF: Vascular endothelial growth factor
VEGFR: Vascular endothelial growth factor receptor.
